# Molecular Remodeling of Left and Right Ventricular Myocardium in Chronic Anthracycline Cardiotoxicity and Post-Treatment Follow Up

**DOI:** 10.1371/journal.pone.0096055

**Published:** 2014-05-07

**Authors:** Olga Lenčová-Popelová, Eduard Jirkovský, Yvona Mazurová, Juraj Lenčo, Michaela Adamcová, Tomáš Šimůnek, Vladimír Geršl, Martin Štěrba

**Affiliations:** 1 Department of Pharmacology, Faculty of Medicine in Hradec Králové, Charles University in Prague, Hradec Králové, Czech Republic; 2 Department of Histology and Embryology, Faculty of Medicine in Hradec Králové, Charles University in Prague, Hradec Králové, Czech Republic; 3 Institute of Molecular Pathology, Faculty of Military Health Sciences, University of Defence, Hradec Králové, Czech Republic; 4 Department of Biochemical Sciences, Faculty of Pharmacy in Hradec Králové, Charles University in Prague, Hradec Králové, Czech Republic; 5 Department of Physiology, Faculty of Medicine in Hradec Králové, Charles University in Prague, Hradec Králové, Czech Republic; Emory University, United States of America

## Abstract

Chronic anthracycline cardiotoxicity is a serious clinical issue with well characterized functional and histopathological hallmarks. However, molecular determinants of the toxic damage and associated myocardial remodeling remain to be established. Furthermore, details on the different propensity of the left and right ventricle (LV and RV, respectively) to the cardiotoxicity development are unknown. Hence, the aim of the investigation was to study molecular changes associated with remodeling of the LV and RV in chronic anthracycline cardiotoxicity and post-treatment follow up. The cardiotoxicity was induced in rabbits with daunorubicin (3 mg/kg/week for 10 weeks) and animals were sacrificed either at the end of the treatment or after an additional 10 weeks. Daunorubicin induced severe and irreversible cardiotoxicity associated with LV dysfunction and typical morphological alterations, whereas the myocardium of the RV showed only mild changes. Both ventricles also showed different expression of ANP after daunorubicin treatment. Daunorubicin impaired the expression of several sarcomeric proteins in the LV, which was not the case of the RV. In particular, a significant drop was found in titin and thick filament proteins at both mRNA and protein level and this might be connected with persistent LV down-regulation of GATA-4. In addition, the LV was more affected by treatment-induced perturbations in calcium handling proteins. LV cardiomyocytes showed marked up-regulation of desmin after the treatment and vimentin was mainly induced in LV fibroblasts, whereas only weaker changes were observed in the RV. Remodeling of extracellular matrix was almost exclusively found in the LV with particular induction of collagen I and IV. Hence, the present study describes profound molecular remodeling of myocytes, non-myocyte cells and extracellular matrix in response to chronic anthracycline treatment with marked asymmetry between LV and RV.

## Introduction

Anthracycline antibiotics (ANT, *e.g.*, doxorubicin, epirubicin or daunorubicin) rank among the most effective anticancer drugs, but their clinical use has been hampered by the risk of cardiotoxicity. Although several types of ANT cardiotoxicity have been recognized, the most important are chronic forms associated with dilated cardiomyopathy and heart failure [1], [2]. They develop within the first year from ANT exposure (early onset form) or with a delay of several years (late onset form) and both are largely irreversible [2]. The chronic cardiotoxicity is common to all ANT derivatives introduced into the clinical practice and the severity of the toxic damage depends on the lifetime cumulative dose of the drug, although significant inter-individual variability has been reported [2], [3].

The free left ventricular (LV) wall and interventricular septum are particularly prone to damage by chronic ANT cardiotoxicity, whereas the right ventricular (RV) myocardium is reported to be significantly less affected [4]. It is noteworthy that a sound biological explanation for this apparent asymmetry is missing. At the histopathological level degenerative changes in cardiomyocytes are usually found in multiple foci throughout the affected myocardium and are typically characterized as a disarray/loss of myofibrils with cytoplasmic vacuolization [5], [6]. Damaged cardiomyocytes undergo profound pathological remodeling and often contain remnants of myofibrils, particularly only scattered clumps of Z-band material, along with the abnormal mitochondria [7], [8]. The injury may progress towards cardiomyocyte death which is followed by the replacement fibrosis. [5], [6]. Different ANT derivatives have been demonstrated to induce practically indistinguishable histopathological hallmarks when administered in repeated cycles to patients as well as to different animal species, including dogs, rabbits or rodents [9].

While functional and histopathological features of chronic ANT cardiotoxicity have been well characterized, the molecular signature of the toxicity and profound myocardial remodeling, which accompanies ANT cardiotoxicity development, is still poorly understood, despite many hypotheses having been proposed [10]–[12]. In this context, it is worth noting that doxorubicin has been shown to suppress an expression of muscle and heart-specific proteins including several sarcomeric ones. These changes have been attributed to a loss of myofibrils which is a prominent feature of ANT toxicity [13], [14]. Several *in vitro* studies also demonstrated ANT-induced impairment of an expression of essential cardiac transcriptional factors GATA-4 and cardiac ankyrin repeat domain (CARP, Ankrd1) [15]–[17]. These events can significantly contribute to the impaired homeostasis of sarcomeric proteins and myofibrillar disarray/loss [18]. The latter morphological feature was also associated with calpain-dependent cleavage of titin, a giant protein acting as the molecular spring within the sarcomere [19]. Others have highlighted ANT-induced activation of ubiquitin-proteasome system [20] which is responsible for targeted degradation of proteins and maintenance of protein quality control in adult cardiomyocytes. Besides cardiomyocytes, molecular and functional remodeling in response to ANT cardiotoxicity certainly involves other myocardial cells and extracellular matrix. In contrast to other types of cardiomyopathy (*e.g.,* ischemic), the details of this process are not well described. Hence, despite multiple isolated observations an insight into the molecular basis of chronic ANT cardiotoxicity and associated myocardial remodeling is still rather limited. Furthermore, the majority of studies performed so far used acute or subacute cardiotoxicity protocols and focused only on the LV, while changes in RV remain to be determined.

The aim of the present investigation was to study molecular changes associated with the remodeling of the LV and RV in response to chronic ANT cardiotoxicity induction and post-treatment follow up.

## Materials and Methods

### Animals and Experimental Design

This study was carried out in accordance with the recommendations of the Guide for the Care and Use of Laboratory Animals of the National Academy of Sciences [21]. The protocol was approved by the internal Animal Welfare Body of the Faculty of Medicine in Hradec Králové, Charles University in Prague (Permit Number: 15254/2011–30). The cardiotoxicity was induced in a well-established schedule [22], [23] in male Chinchilla rabbits (n = 32) by repeated administration of daunorubicin (DAU, 3 mg/kg i.v., n = 16, Daunoblastina, Pfizer, Rome, Italy) once weekly for ten weeks, whereas animals in the control group received saline (1 mL/kg i.v., n = 16) in the same schedule. A week after the last administration (*i.e.*, at the end of the treatment period), the animals in control and DAU group were randomized to sacrifice (n = 8 in each group) or to follow up for additional 10 weeks (follow up period, n = 8 in each group). In the follow up period LV fractional shortening lower than 20% was taken as evidence for severe (decompensated) heart failure and these animals were prematurely sacrificed to avoid loss of myocardial samples due to the sudden death. All experimental non-invasive procedures were performed under anesthesia containing ketamine (30 mg/kg, i.m., Narketan, Vétoquinol, Ittigen, Switzerland) and midazolam (1.25 mg/kg, i.m., Midazolam Torrex, Chiesi Pharmaceuticals, Vienna, Austria), while freshly prepared pentobarbital solution (4% (w/w), Sigma, St. Louis, MO) was used for intravenous anesthesia during final invasive hemodynamic measurements and for animal overdose at the end of the experiment. All efforts were made to minimize the suffering of the animals.

### Examination of Cardiac Functions

Echocardiography (Vivid 4 equipped with a 10 MHz probe, GE Medical Systems Ultrasound, Hatfield, United Kingdom) examination was performed weekly from the 9^th^ week until the end of the study. All measurements were performed from guided M-mode examination at the tips of the mitral valve in the long and short axis view. LV diastolic and systolic dimensions were determined and fractional shortening was calculated as an index of the systolic function from both parameters. At the end of the experiment, the invasive hemodynamic examination of the LV was performed in pentobarbital anesthesia using Mikro-Tip pressure catheter (2.3F, Millar Instruments, Houston, TX) connected to a data acquisition system (Powerlab, ADInstruments, Bella Vista NSW, Australia). The Chart 5.4.2 software (ADInstruments, Bella Vista NSW, Australia) was used for data analysis and calculation of the maximum rate of LV isovolumic pressure rise (index dP/dt_max_) and the maximum rate of LV isovolumic pressure decline (dP/dt_min_). The measurement was performed after the 15-min equilibration interval to stabilize the animal after the preparation.

### Heart Harvesting and Myocardial Sample Preparation

At autopsy, the heart was rapidly excised, washed and briefly retrogradely perfused with ice-cold saline. After drying with a tissue, the heart was weighed and transverse sections of the LV and RV were taken for histological examinations. The whole rest of the free LV and RV wall was pulverized in liquid nitrogen and stored at −80°C.

### Histology and Immunohistochemistry

Hearts were transversely cut through both ventricles closely under the atrioventricular orifices and fixed in 4% neutral formaldehyde for 3 days. Routinely prepared serial paraffin sections (6 µm thick) were stained with hematoxylin and eosin. Masson’s blue trichrome and Picrosirius red staining were used for detection of collagen fibers. Briefly, deparaffinized and rehydrated sections were first stained with Weigert’s hematoxylin (nuclear staining). In case of Masson’s blue trichrome, slides were stained with Ponceau 2R/Acid Fuchsin solution and Aniline Blue solution. For Picrosirius red staining, sections were incubated in 0.1% (w/v) Sirius Red F3B (Sigma, St. Louis, MO) in saturated aqueous solution of picric acid. Finally, sections from both procedures were dehydrated using increasing concentrations of ethanol followed by immersion in xylol and were eventually mounted in DPX medium (Sigma, St. Louis, MO).

For immunohistochemical analyses the following mouse monoclonal antibodies were used: anti-desmin (clone 33, Bio-Genex, Fremont, CA; dilution 1∶40), anti-vimentin (clone V9, Sigma, St. Louis, MO; dilution 1∶100) and anti-ubiquitin (clone Ubi-1, Hycult Biotechnologies, Uden, Netherlands; dilution 1∶40). The sections were processed using peroxidase anti-peroxidase immunohistochemistry. Briefly, deparaffinized and rehydrated sections were pre-incubated with an aqueous solution of hydrogen peroxide (0.6%) for 20 minutes to inactivate endogenous peroxidase. For the detection of vimentin, the heat-induced epitope retrieval (HIER) technique was carried out in citrate buffer (pH 6.0). Incubation with primary monoclonal antibodies was performed overnight at 4°C. The sections were then incubated with biotinylated secondary antibody (Jackson ImmunoResearch Lab., West Grove, PA) and subsequently with horseradish peroxidase-labeled streptavidin solution (Dako, Glostrup, Denmark) both for 45 minutes at room temperature. Visualization was made with 3,3′-diaminobenzidine-tetrahydrochloride (DAB, Sigma, St. Louis, MO). With an exception of ubiquitin detection, sections were counterstained with Gill’s hematoxylin. Photomicrographs were made using the Olympus BX51 microscope with Cybernetics software version 4.51 (Laboratory Imaging, Prague, Czech Republic).

### RNA Isolation and Quantitative Real-time PCR Analyses

Total RNA was isolated from the LV and RV myocardium of all animals in the study using TRIzol reagent (Sigma, St. Louis, MO). One microgram of total RNA of each sample was reverse transcribed using a High Capacity cDNA Reverse Transcription Kit (Applied Biosystems, Foster City, CA). Quantitative real-time PCR was performed on a 7500HT Fast Real-Time PCR System (Applied Biosystems, Foster City, CA) with TaqMan Fast Universal PCR Master Mix (Applied Biosystems, Foster City, CA) under amplification conditions: 95°C for 3 min, and 50 cycles of each 95°C for 5 s and 60°C for 25 s. Each cDNA sample was analyzed in triplicate and the mean threshold cycle values were transformed into relative expression using the Pfaffl method [24]. Commercial gene expression assays (Table S1) were obtained from Generi Biotech (Hradec Králové, Czech Republic) or Applied Biosystems (Foster City, CA). The expression data were normalized by *HPRT1* expression.

### Western Blotting

The LV and RV myocardial samples were sonicated in ice-cold RIPA buffer (Sigma, St. Louis, MO) with 10 mM N-ethylmaleimide and protein inhibitor solution (Complete Protease Inhibitor Cocktail, Roche Diagnostics, Mannheim, Germany). After centrifugation (10 000×g for 15 min, 4°C), the supernatants were collected and 10, 2.5 or 0.5 µg of total protein from each sample were mixed with loading buffer under reducing conditions and separated by SDS-PAGE using Any kD or 10% Mini-PROTEAN TGX Precast Gels (Bio-Rad, Hercules, CA). Following electrophoresis, the proteins were transferred to PVDF membranes. After blocking with 5% non-fat milk in TBS containing Tween 20, the membranes were incubated overnight at 4°C with primary antibodies against α-actin (Alpha-Sr-1, Dako, Glostrup, Denmark; dilution 1∶2000), desmin (D33, Dako, Glostrup, Denmark; dilution 1∶2000), myosin regulatory light chain 2 (ventricular form) (Acris Antibodies, San Diego, CA; dilution 1∶500), NCX (6H2, Thermo Scientific, Rockford, IL; dilution 1∶5000), SERCA2 (IID8, Thermo Scientific, Rockford, IL; dilution 1∶1000), ubiquitin (Ubi-1, Hycult Biotechnologies, Uden, Netherlands; dilution 1∶10) and vimentin (V9, Sigma, St. Louis, MO; dilution 1∶7500). The membranes were incubated with anti-mouse or anti-goat secondary antibodies conjugated with horseradish peroxidase (Dako, Glostrup, Denmark) for 1 hour at room temperature. Membranes were developed using BM Chemiluminescence Blotting Substrate (Roche Diagnostics, Mannheim, Germany) and exposed to X-ray film. Densitometric quantitation of results was performed using Quantity One software (Bio-Rad, Hercules, CA). To ensure equal loading of proteins, membranes were probed for GAPDH (Sigma, St. Louis, MO). The western blot analyses included all samples from the study (n = 8 in each group).

### Quantitation of Titin and Myosin Heavy Chains

The pulverized LV and RV myocardium (from all studied animals) were homogenized in ice-cold buffer containing 8 M urea, 1% (w/v) sodium deoxycholate and protein inhibitor solution (Complete Protease Inhibitor Cocktail, Roche Diagnostics, Mannheim, Germany) with the addition of glass beads. After centrifugation (14 000×g for 10 min, 10°C), the supernatants were collected and used for detection of titin and myosin heavy chains (MHC) according to Tatsumi *et al.* [25] with minor modification. To determine MHC, 10 µg of total protein from each sample were mixed with Laemmli buffer under reducing conditions (10 mM TCEP, Sigma, St. Louis, MO) and lanes were loaded on a 2.8% acrylamide gels strengthened with 0.75% agarose. For titin detection, 80 µg of total protein were mixed with loading buffer containing 5% β-mercaptoethanol (Bio-Rad, Hercules, CA) and analyzed by SDS-PAGE using a 2% acrylamide gels strengthened with 0.75% agarose. Electrophoresis was performed at 1.5 mA/gel. After the separation, the gels were stained with SYPRO Ruby Protein Gel Stain (Bio-Rad, Hercules, CA) for quantification or with Coomassie blue for mass spectrometry identification of the proteins (see Method S1). Gels were wet scanned using Molecular Imager PharosFX System (Bio-Rad, Hercules, CA). T1 titin (both N2BA and N2B isoforms), titin degradation product T2 and MHC were analyzed using Quantity One software (Bio-Rad, Hercules, CA). Protein loading was controlled with anti-GAPDH antibody (Sigma, St. Louis, MO) (see above).

### Cardiac Troponin T Determinations

Cardiac troponin T was determined in plasma and tissue samples using Elecsys Tropo T hs (Roche Diagnostics, Basel, Switzerland) on the Elecsys 2010 analyzer (Hitachi High-Technologies Corporation, Tokyo, Japan) according to the manufacturer’s recommendations. A limit of detection was 0.003 µg/L. Blood was sampled before the 1^st^, 5^th^, 8^th^, and 10^th^ drug administration, a week after the last drug administration and weekly in the follow up period. Area under curve (AUC) of plasma troponin T concentrations was determined using GraphPad Prism 5.0 (GraphPad Software, La Jolla, CA). Cardiac troponin T in myocardial samples was determined in the supernatant prepared for western blotting after appropriate dilution with saline (see above).

### Biochemical Determination of Collagenous Proteins

The collagenous proteins in the LV and RV myocardium were estimated from myocardial hydroxyproline concentration. In brief; pulverized myocardial samples were weighed, dried and hydrolyzed in hydrochloride acid (6 M) for 3 hours at 130°C. Then, oxidation of the hydrolyzed samples was started with the addition of chloramine T in acetate-citrate buffer, pH 6.0. The mixture was incubated for 20 minutes at room temperature and the reaction was stopped by adding 20 volume of Ehrlich's reagent solution. After incubation of samples at 65°C for 15 minutes, the hydroxyproline concentration was measured spectrophotometrically at 550 nm [26]. The results were determined as mg/g of wet tissue weight and then compared over the control group in the treatment period.

### Determinations of Protein Concentrations

BCA Protein Assay Kit (Sigma, St. Louis, MO) was used for determination of protein concentrations in the tissue lysates.

### Statistical Analyses

All statistical analyses were performed using statistical software SigmaStat 3.5 (SPSS, Chicago, IL). The data are presented as means ± SD or median along with the interquartile range according to the data distribution character and statistical significance was determined using either One Way ANOVA for continuous variables or One Way ANOVA on Ranks for nonparametric variables. Differences were considered statistically significant at *P*<0.05.

## Results

### Cardiac Function and General Toxicity

**Figure 1:**
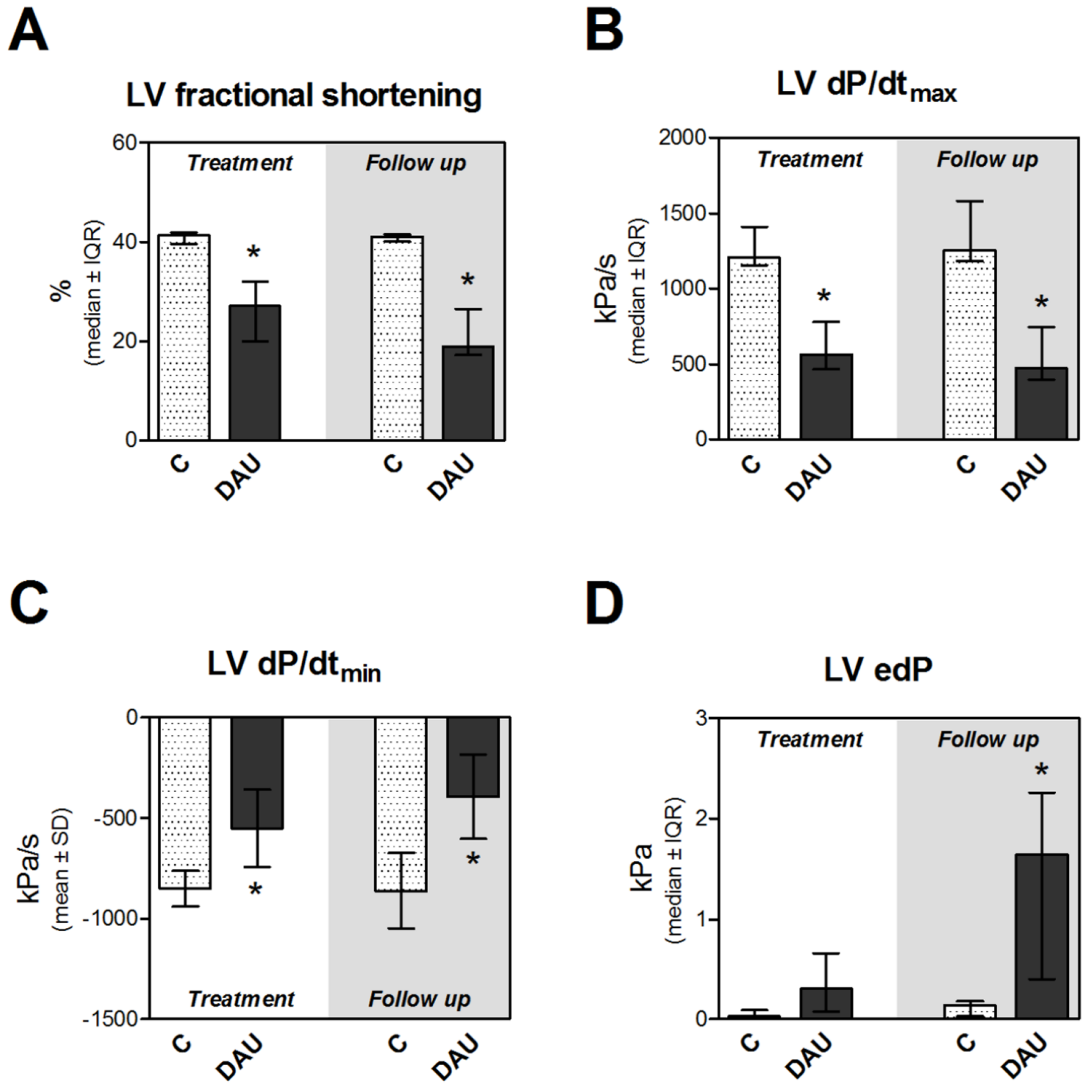
Cardiac function in chronic anthracycline cardiotoxicity and post-treatment follow up. **A**, Echocardiographic examination of left ventricular systolic function (LV fractional shortening, last measured value). Invasive examination of left ventricular systolic function (index dP/dt_max_, **B**), left ventricular diastolic function (index dP/dt_min_, **C**) and left ventricular end-diastolic pressure (LV edP, **D**). Statistical significances (One Way ANOVA/One Way ANOVA on Ranks according to data character, *P*<0.05) within each study period (*). C - control group, DAU - daunorubicin group.

**Figure 2:**
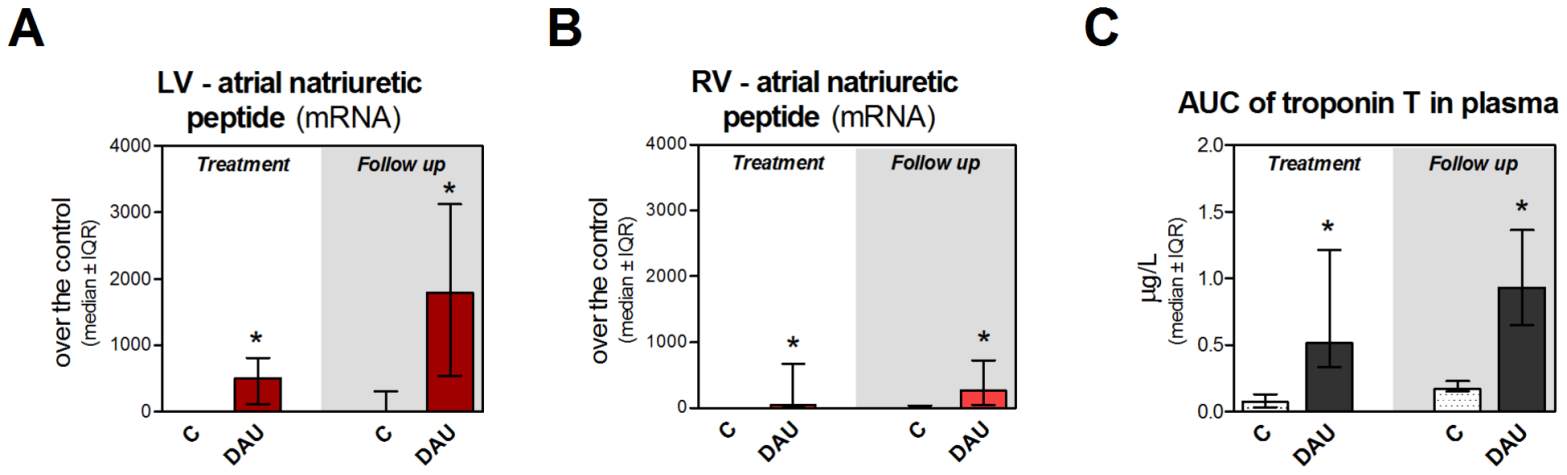
Markers of cardiac damage and myocardial dysfunction in chronic anthracycline cardiotoxicity and post-treatment follow. Expression of atrial natriuretic peptide in the left (**A**) and right (**B**) ventricle. **C**, Area under curve of plasma concentrations of cardiac troponin T determined by hypersensitive ELISA. Statistical significances (One Way ANOVA on Ranks, *P*<0.05) within each study period (*). C - control group, DAU - daunorubicin group.

Chronic DAU treatment in rabbits induced significant LV systolic dysfunction as documented by both echocardiographic and invasive examinations of cardiac function (Fig. 1A and Fig. 1B, respectively). As evident from these measurements, the systolic dysfunction did not improve in the post-treatment period. Instead, it was persisting or slightly worsening. Similar changes due to the DAU treatment were also documented in the LV diastolic function (Fig. 1C). Although there was a tendency towards a raise of the LV end-diastolic pressure at the end of the DAU treatment, it was markedly and significantly elevated in the post-treatment follow up (Fig. 1D) which further confirms the severity of the LV dysfunction at this stage. Significant induction of ANP gene expression was found in both ventricles after the DAU treatment, but it was apparently more distinct in the LV myocardium (Fig. 2A and Fig. 2B).

The treatment also induced a raise of cardiac troponin T in plasma as compared to relevant controls (Fig. 2C). A heart to body weight ratio showed insignificant tendency towards increase at the end of the treatment, while statistical difference was found in the follow up period (Fig. S1). In contrast to the control group, a moderate premature mortality occurred in the treatment period of the DAU group (two out of eight, between 9^th^–11^th^ weeks), while in the post-treatment period, a progression of the systolic dysfunction scheduled six animals to be sacrificed according to the study protocol. During autopsy and necropsy, LV dilation was frequently noted in animals from DAU-treated group, particularly in the follow up period, whereas RV dilation was found only in the animals with the end-stage heart failure. Pleural effusions and abdominal fluid were found in prematurely dead/sacrificed animals, while these signs were completely absent in controls.

### Histological Examinations of the Myocardial Morphology

**Figure 3:**
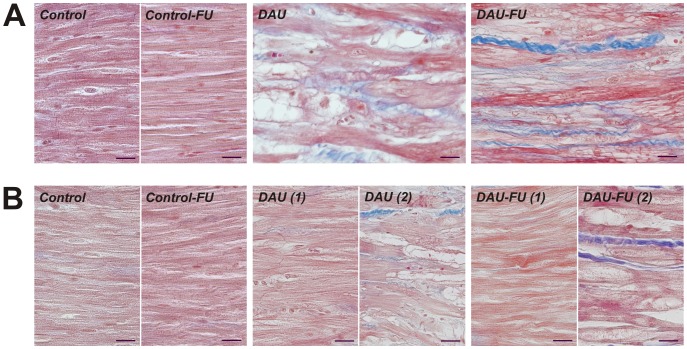
Morphology of the left and right ventricle in chronic anthracycline cardiotoxicity and post-treatment follow up. **A,** Left ventricle, **B,** right ventricle. Masson’s blue trichrome; collagen fibers stained in blue. Scale bar - 20 µm. FU - follow up, DAU - daunorubicin group, DAU(1) and DAU-FU(1) - typical (almost normal) morphology in the RV of DAU-treated animals without severe congestion of the lungs, DAU(2) and DAU-FU(2) - pathological changes in the RV of DAU-treated animals with severe left-sided heart failure accompanied by severe congestion of the lungs.

Histological examination of the myocardium from the DAU group showed focal myocardial damage spread throughout the LV free wall and interventricular septum (Fig. 3A). The particularly severe damage was usually found around large coronary arteries - primarily left coronary artery, *i.e.,* in the anterior longitudinal sulcus, less in relation to the posterior interventricular branch of right coronary artery - and in the interventricular septum. Morphological changes in affected cardiomyocytes primarily comprised different degrees of myofibrillar loss and cytoplasmic vacuolization. Degenerating cardiomyocytes appeared enlarged, often contained remnants of myofibrils only along with the different amounts of vacuoles whose volume evidently enlarged with the progression of degenerative process. Dead myocytes were continuously replaced by fibrotic connective tissue and focal replacement fibrosis developed. The degenerative changes in cardiomyocytes were persistent even in animals surviving until the end of the 10-week follow up. However, the changes tended to be less frequent in animals that survived longer in the post-treatment follow up, whilst the replacement fibrosis became more prominent and the connective tissue tended to be organized into fine or more conspicuous scars.

In contrast to significant degenerative changes found in the LV myocardium and interventricular septum, the RV myocardium was strikingly less affected by the DAU treatment. Degenerative changes were markedly less frequent and rather mild. In many cases almost normal structure of the RV myocardium was observed (Fig. 3B). Larger foci of degenerating cardiomyocytes in the RV myocardium were almost exclusively found in animals suffering from severe global heart failure. This was associated with lung congestion and pleural effusion along with the RV dilation which may suggest the effect of pressure overloading. In such cases, the RV myocardium was most affected in the parts of wall adjacent to the interventricular septum, while the central part of RV wall often showed relatively mild changes only. A similar morphological picture was also found in the follow up period.

### Expression of Sarcomeric Proteins and Myocardial Transcription Factors

**Figure 4:**
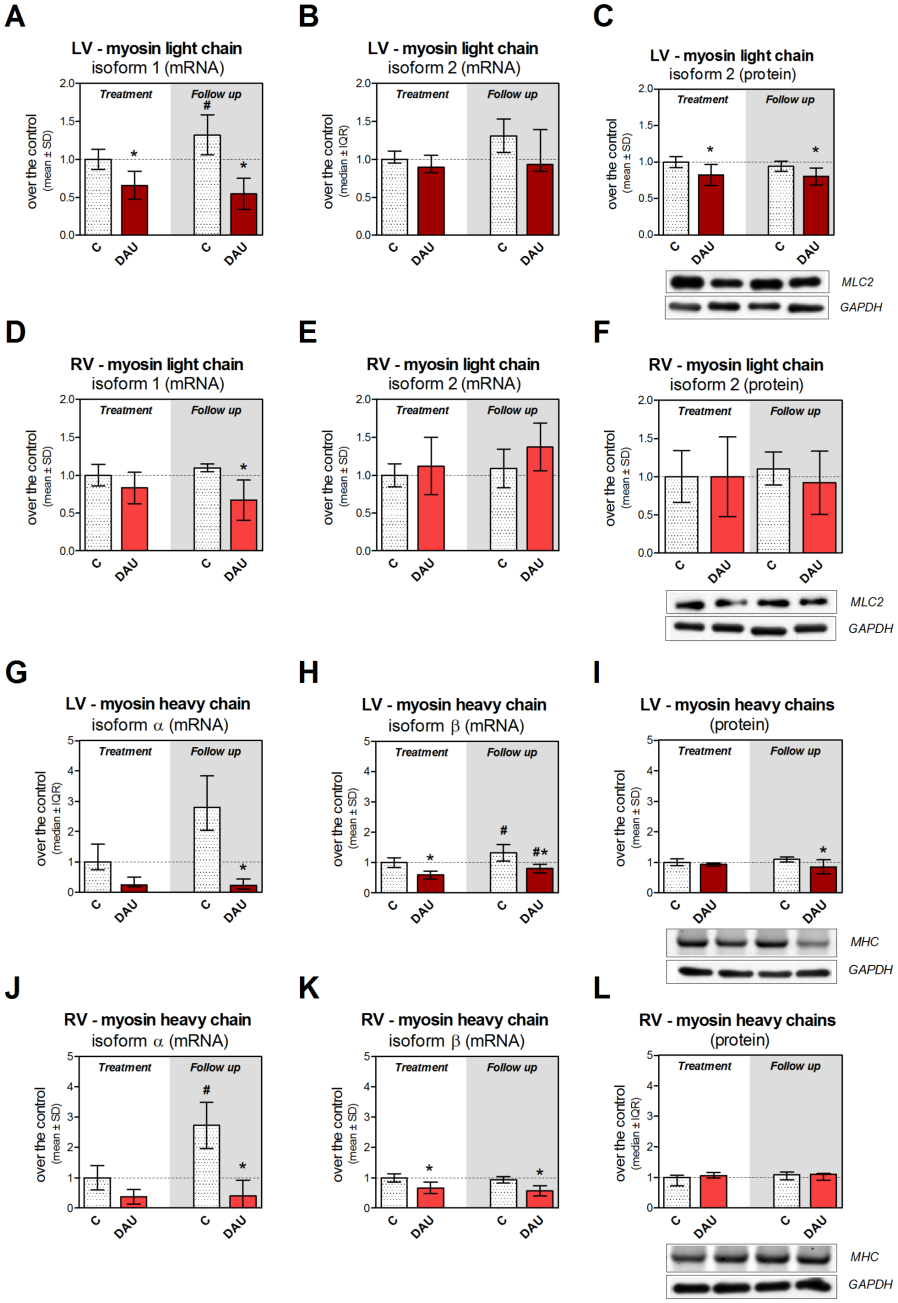
Expression of light and heavy myosin chains in chronic anthracycline cardiotoxicity and post-treatment follow up. Gene expression of cardiac myosin light chain isoform 1 (**A**, **D**) and isoform 2 (**B**, **E**) in the left and right ventricular myocardium, respectively. Western blot analysis of myosin light chain isoform 2 in the left (**C**) and right (**F**) ventricular myocardium. Gene expression of myosin heavy chain isoform α (**G**, **J**) and isoform β (**H**, **K**) in the left and right ventricular myocardium, respectively. Quantification of myosin heavy chains in the left (**I**) and right (**L**) ventricular myocardium. SYPRO Ruby stained 2.8% polyacrylamide gel strengthen with 0.75% agarose. Statistical significances (One Way ANOVA/One Way ANOVA on Ranks according to data character, *P*<0.05) within each study period (*) or between the treatment and post-treatment period (#). C - control group, DAU - daunorubicin group.

First, we investigated the impact of the DAU treatment on the expression of genes encoding thick filament proteins, *i.e.,* myosin light and heavy chains. The qRT-PCR analysis revealed transcriptional down-regulation of cardiac myosin light chain isoform 1 (cMLC1) in the LV in both study intervals (Fig. 4A), while in the RV this change was only significant in the follow up period (Fig. 4D). The same analysis of the myosin regulatory light chain 2 (MLC2v) found no significant change in the myocardium of both ventricles (Fig. 4B and Fig. 4E). Western blot analysis confirmed a slight but significant drop in the abundance of this protein in the LV in both studied intervals (Fig. 4C). No change was found in the RV (Fig. 4F). The expression of the myosin heavy chain alpha isoform (αMHC) was rather variable with a tendency to increase in the control group in the follow up period, while there was an opposite tendency in DAU-treated animals (Fig. 4G and Fig. 4J). The expression of major isoform of myosin heavy chains (βMHC) showed significant down-regulation due to the DAU treatment in both LV and RV myocardium (Fig. 4H and Fig. 4K). A very slight but significant increase of expression of this protein was found in the LV during the follow up period in both DAU- and saline-treated animals as compared to end-of-treatment values, while no such change was observed in the RV myocardium. The SDS electrophoresis followed by fluorescent detection and mass spectrometry identification of the proteins (Method S1 and Table S2) revealed decreased MHC levels in the LV after the DAU treatment in the follow up period only (Fig. 4I). No changes were observed in the RV (Fig. 4L).

**Figure 5:**
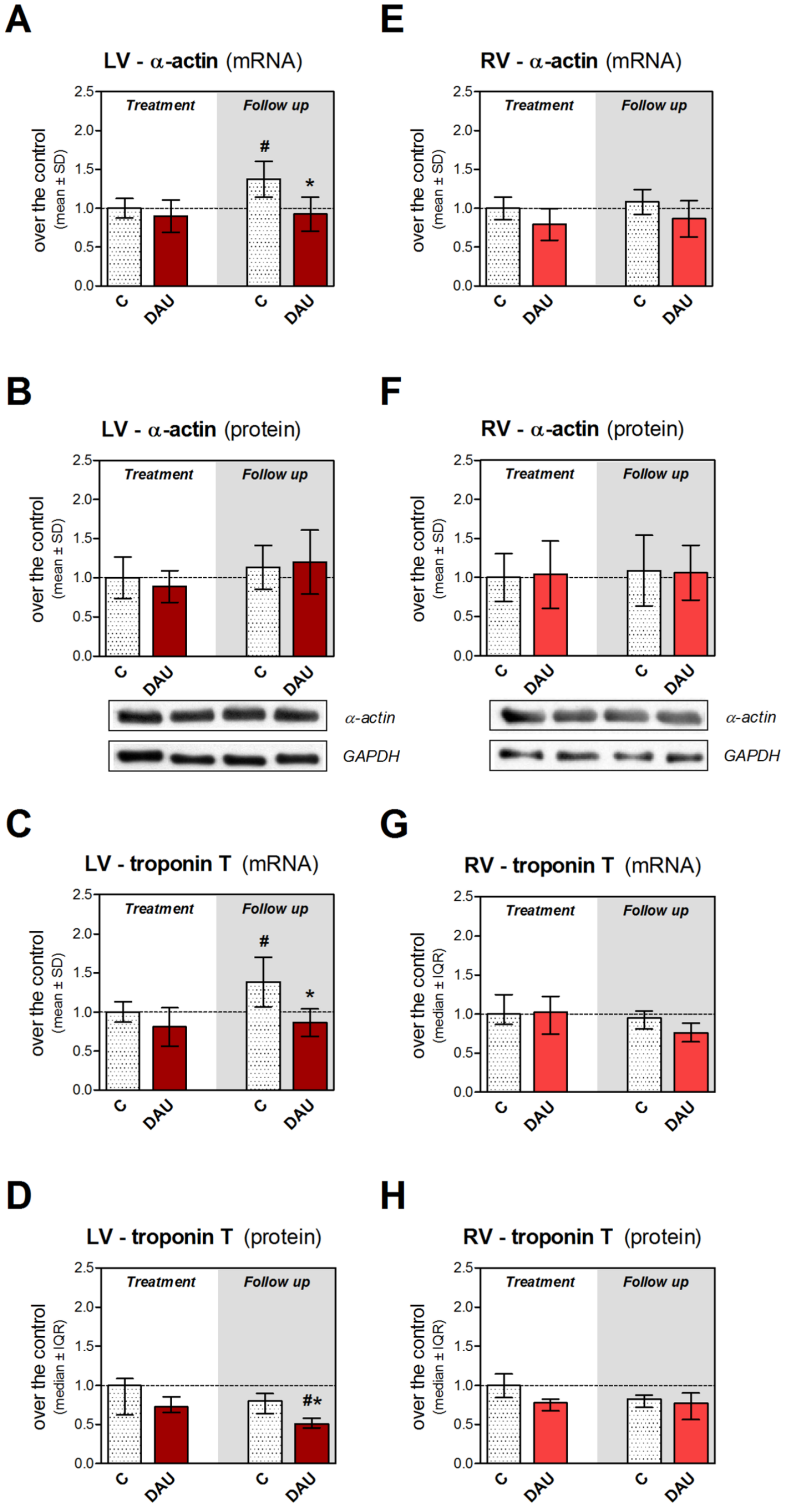
Expression of thin filament proteins in chronic anthracycline cardiotoxicity and post-treatment follow up. Gene expression of cardiac α-actin (**A**, **E**) and cardiac troponin T (**C**, **G**) in the left and right ventricular myocardium, respectively. Western blot analysis of protein levels of cardiac α-actin in the left (**B**) and right (**F**) ventricle. ELISA determined abundance of the cardiac troponin T in the left (**D**) and right (**H**) ventricular myocardium. Statistical significances (One Way ANOVA/One Way ANOVA on Ranks according to data character, *P*<0.05) within each study period (*) or between the treatment and post-treatment period (#). C - control group, DAU - daunorubicin group.

Despite the well-developed cardiomyopathy, the analysis of gene expression of cardiac α-actin and cardiac troponin T in the LV showed no significant change at the end of the DAU treatment, but moderate difference was found in the post-treatment period (Fig. 5A and Fig. 5C, respectively). The latter finding was likely co-determined by the relatively mild, but significant increase in the corresponding control group which was not followed by the DAU group. A similar trend was also found in tropomyosin expression (Fig. S2a). While protein levels of troponin T in the LV showed a drop in the DAU group in the post-treatment period (without any tendency to change in the control group, Fig. 5D), no difference was found in cardiac α-actin (Fig. 5B). Protein as well as mRNA levels of these thin filament molecules were not significantly changed in the RV (Fig. 5E–H and Fig. S2b).

**Figure 6:**
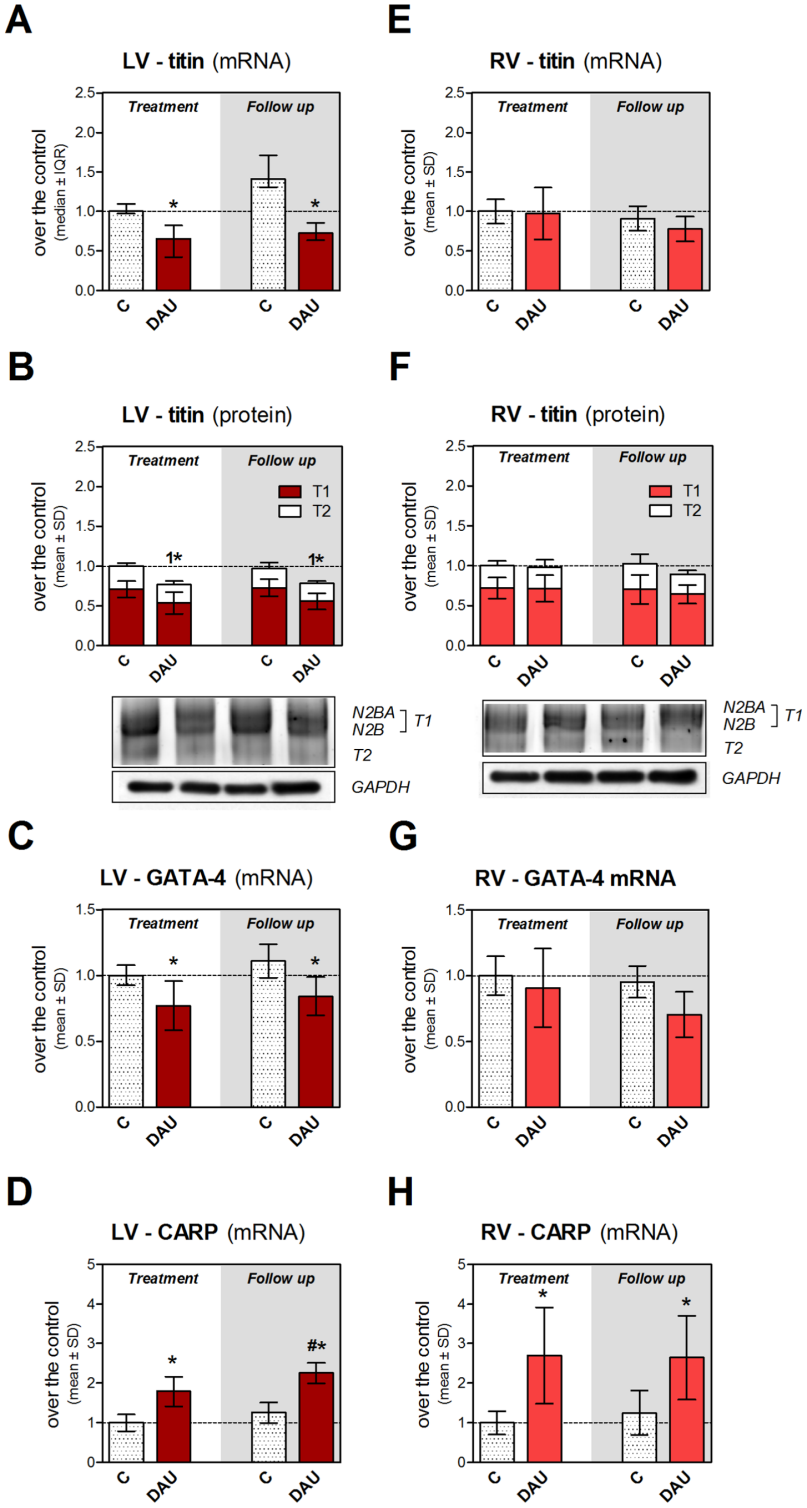
Expression of titin and cardiac transcriptional factors in chronic anthracycline cardiotoxicity and post-treatment follow up. Gene expression of titin in the left (**A**) and right (**E**) ventricular myocardium. Quantification of titin isoforms (top T1 band and bottom T2 band) in the left (**B**) and right (**F**) ventricular myocardium. SYPRO Ruby stained 2% polyacrylamide gel strengthen with 0.75% agarose. Gene expression of cardiac transcriptional factors GATA-4 (**C**, **G**) and cardiac ankyrin repeat protein (CARP, Ankrd1, **D**, **H**) in the left and right ventricular myocardium, respectively. Statistical significances (One Way ANOVA/One Way ANOVA on Ranks according to data character, *P*<0.05) within each study period (*) or between the treatment and post-treatment period (#) and within each study period in abundance of T1 titin isoform (**1**). C - control group, DAU - daunorubicin group.

Gene expression of titin showed marked down-regulation due to the treatment in the LV at the end of the treatment period and this change persisted in the follow up period (Fig. 6A). SDS electrophoresis separation of this protein followed by its mass spectrometry identification (Method S1 and Table S2) confirmed a decrease of titin abundance in the LV myocardium due to the treatment in both studied periods (Fig. 6B). The drop in protein abundance of T1 titin isoform was not accompanied by significant alteration in N2BA/N2B ratio (data not shown) or by increase of titin degradation product T2. It is noteworthy that no change in titin expression was found in the RV after the treatment (Fig. 6E and Fig. 6F). To reveal potential regulation of expression of sarcomeric proteins, we have also analyzed gene expression of important cardiac transcriptional factors GATA-4 and CARP. Interestingly, inverse regulations of expression of these proteins were found. While GATA-4 expression showed persisting down-regulation in the LV (Fig. 6C), CARP expression was markedly induced due to the DAU treatment (Fig. 6D). In contrast, no change in GATA-4 expression was observed in the RV (Fig. 6G), although CARP expression was up-regulated here in similar degree as in the LV (Fig. 6H).

### Changes in Calcium Handling Proteins

**Figure 7:**
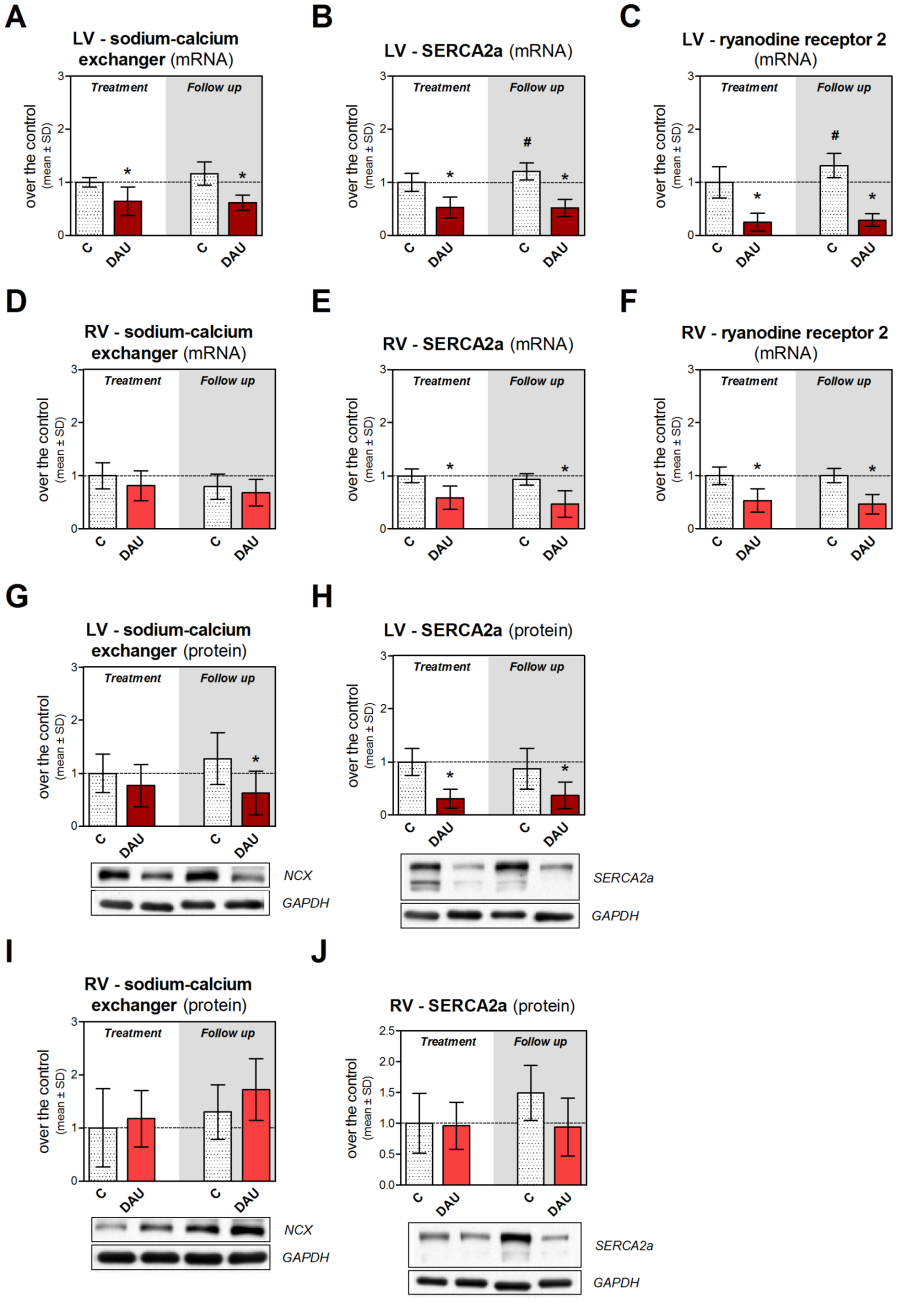
Changes in expression of calcium handling proteins in chronic anthracycline cardiotoxicity and post-treatment follow up. Gene expression of sodium-calcium exchanger (**A**, **D**), SERCA2a (**B**, **E**) and ryanodine receptor 2 **(C**, **F)** in the left and right ventricular myocardium, respectively. Western blot analysis of protein levels of sodium-calcium exchanger (**G**, **I**) and SERCA2a (**H**, **J**) in the left and right ventricular myocardium, respectively. Statistical significances (One Way ANOVA, *P*<0.05) within each study period (*) or between the treatment and post-treatment period (#). C - control group, DAU - daunorubicin group.

The DAU treatment induced marked and persistent down-regulation of gene expression of sodium-calcium exchanger (NCX), ryanodine receptor 2 (RyR2) and SERCA2a in the LV in both studied periods (Fig. 7A–C) and very similar changes were found in NCX and SERCA2a at the protein level (Fig. 7G and Fig. 7H). Treatment-induced alterations in the RV were less profound (Fig. 7D–F) and often insignificant (*e.g.,* changes in NCX or SERCA2a protein levels, Fig. 7I and Fig. 7J).

### Changes in Intermediate Filaments

**Figure 8:**
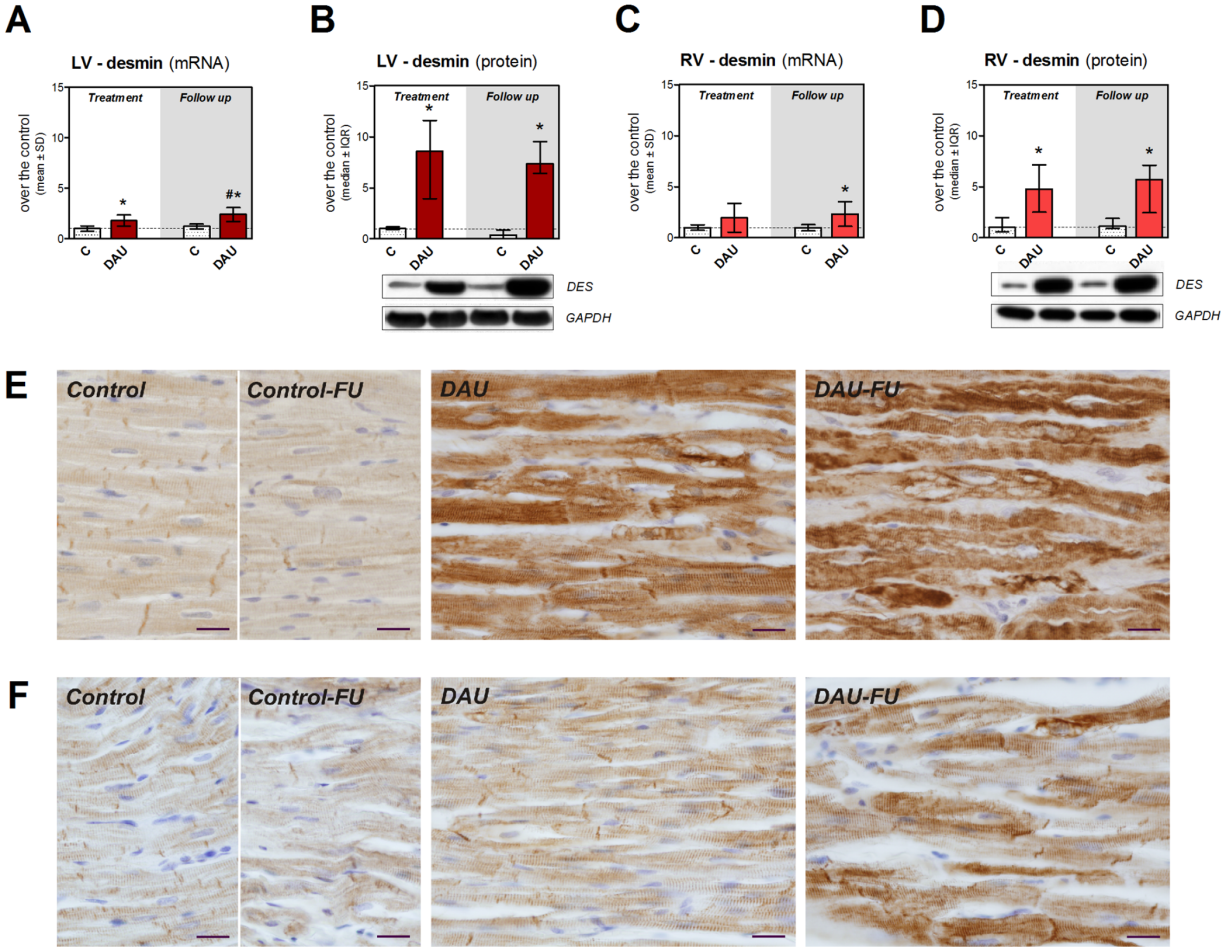
Expression of desmin in chronic anthracycline cardiotoxicity and post-treatment follow up. Expression of desmin at the mRNA (**A**, **C**) and protein (**B**, **D**) level in the left and right ventricular myocardium, respectively. Statistical significances (One Way ANOVA/One Way ANOVA on Ranks according to data character, *P*<0.05) within each study period (*) or between the treatment and post-treatment period (#). Immunohistochemical detection of desmin (brown) in the left (**E**) and right (**F**) ventricular myocardium. Nuclei counterstained with Gill’s hematoxylin. Scale bar - 20 µm. C - control group, DAU - daunorubicin group, FU - follow up.

Gene expression of desmin was found significantly up-regulated in the LV due to the treatment (2-fold) and this change persisted in the follow up period (Fig. 8A). Western blot analysis of the protein levels revealed even much higher difference over the control group (9-fold) (Fig. 8B). In the RV, the significant increase in gene expression was found only in the post-treatment follow up period (Fig. 8C), while western blot analysis showed moderate (5-fold) increase of the protein levels over corresponding controls (Fig. 8D). Immunohistochemical analysis confirmed markedly increased expression of desmin within the cardiomyocytes of the LV due to the DAU treatment and some degenerating cardiomyocytes showed irregular dense immunopositivity which was even more frequently seen in the post-treatment period (Fig. 8E). The same analysis in the RV showed only slight to moderate increase in the desmin immunopositivity in both studied intervals along with preserved expression pattern (Fig. 8F).

**Figure 9:**
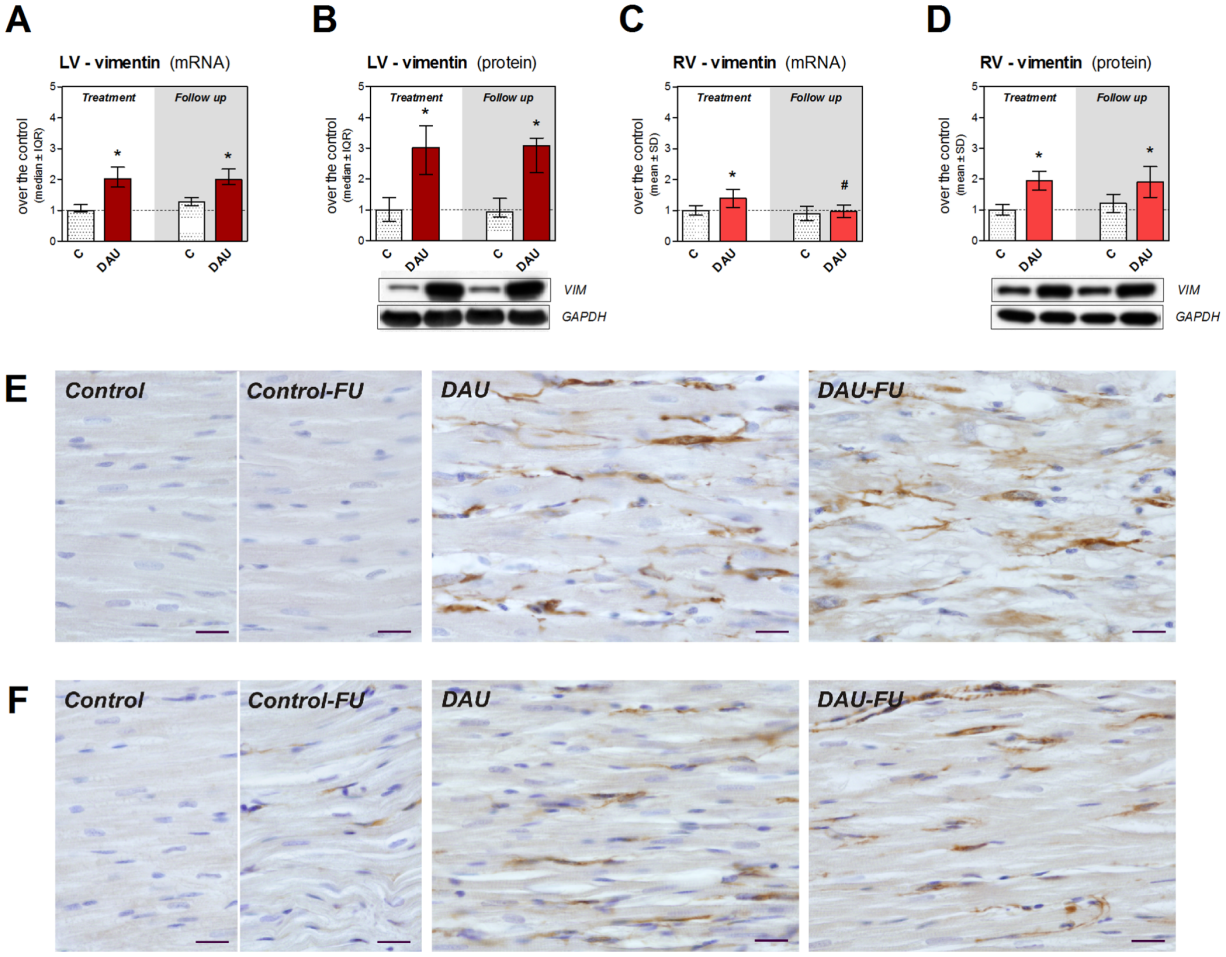
Expression of vimentin in chronic anthracycline cardiotoxicity and post-treatment follow up. Expression of vimentin at the mRNA (**A**, **C**) and protein (**B**, **D**) level in the left and right ventricular myocardium, respectively. Statistical significances (One Way ANOVA/One Way ANOVA on Ranks according to data character, *P*<0.05) within each period (*) or between the treatment and post-treatment period (#). Immunohistochemical detection of vimentin (brown) in the left (**E**) and right (**F**) ventricular myocardium. Nuclei counterstained with Gill’s hematoxylin. Scale bar - 20 µm. C - control group, DAU - daunorubicin group, FU - follow up.

Gene expression of vimentin, a major intermediate filament of non-myocyte cells of the adult myocardium, showed a 2-fold mRNA up-regulation in the LV due to the treatment (Fig. 9A) and an even higher increase was found at the protein level (Fig. 9B). Induction of vimentin expression was less pronounced in the RV myocardium, where mild significant increase was found at the end of the treatment at the mRNA level with almost control-like values in the post-treatment period (Fig. 9C). Moderate increase in the DAU groups was then detected in the RV at the protein level (Fig. 9D). The immunohistochemical analysis of vimentin in the LV myocardium revealed limited immunopositivity in controls (mostly endothelial and smooth muscle cells), while marked immunopositivity attributed mainly to fibroblasts was found in the DAU-treated animals in both study periods (Fig. 9E). In contrast, there were rather less conspicuous difference between the DAU-treated and control groups in the RV and some hearts from the DAU group showed even comparable vimentin immunopositivity as in appropriate controls (Fig. 9F).

### Changes in the Ubiquitin-proteasome System

**Figure 10:**
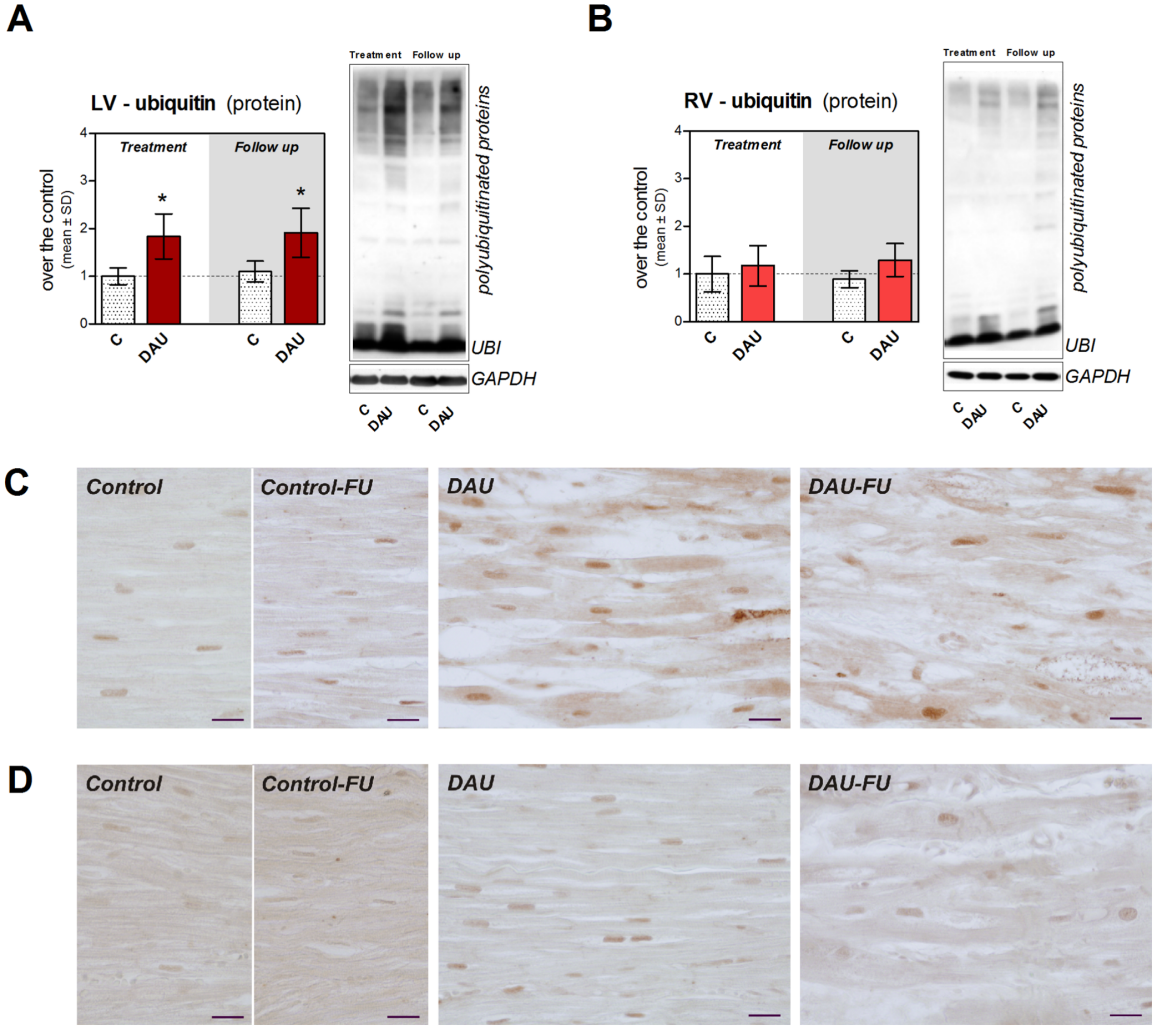
Expression of ubiquitin in chronic anthracycline cardiotoxicity and post-treatment follow up. Western blot analysis of free ubiquitin and polyubiquitinated proteins in the left (**A**) and right (**B**) ventricular myocardium. Statistical significances (One Way ANOVA, *P*<0.05) within each period (*). Immunohistochemical detection of ubiquitin (brown) in the left (**C**) and right (**D**) ventricular myocardium. Scale bar - 20 µm. C - control group, DAU - daunorubicin group, FU - follow up.

While we found increased activity of chymotrypsin, trypsin and PGP-like components of the proteasome system in the LV myocardium to similar extent in both study periods (Fig. S3a–c, respectively), the findings in the RV were less consistent and significant (Fig. S3d–f, respectively). The trypsin-like activity in the LV was the most affected and this change persisted in the follow up period. It was interesting to note that despite the increase in proteasome activity, the DAU treatment was associated with significantly increased abundance of polyubiquitinated proteins in the LV myocardium in both studied intervals (Fig. 10A). However, the same analysis of RV samples showed no change due to the treatment (Fig. 10B). These findings were corroborated by immunohistochemical analysis of ubiquitin in the LV and RV myocardium (Fig. 10C and Fig. 10D, respectively). As compared to controls, more pronounced diffuse ubiquitin immunopositivity was typically found in the LV cardiomyocytes after the DAU treatment, whereas RV cardiomyocytes seemed spared of this effect. The treatment did not induce marked ubiquitin changes in non-myocyte cells.

### Extracellular Matrix Proteins

**Figure 11:**
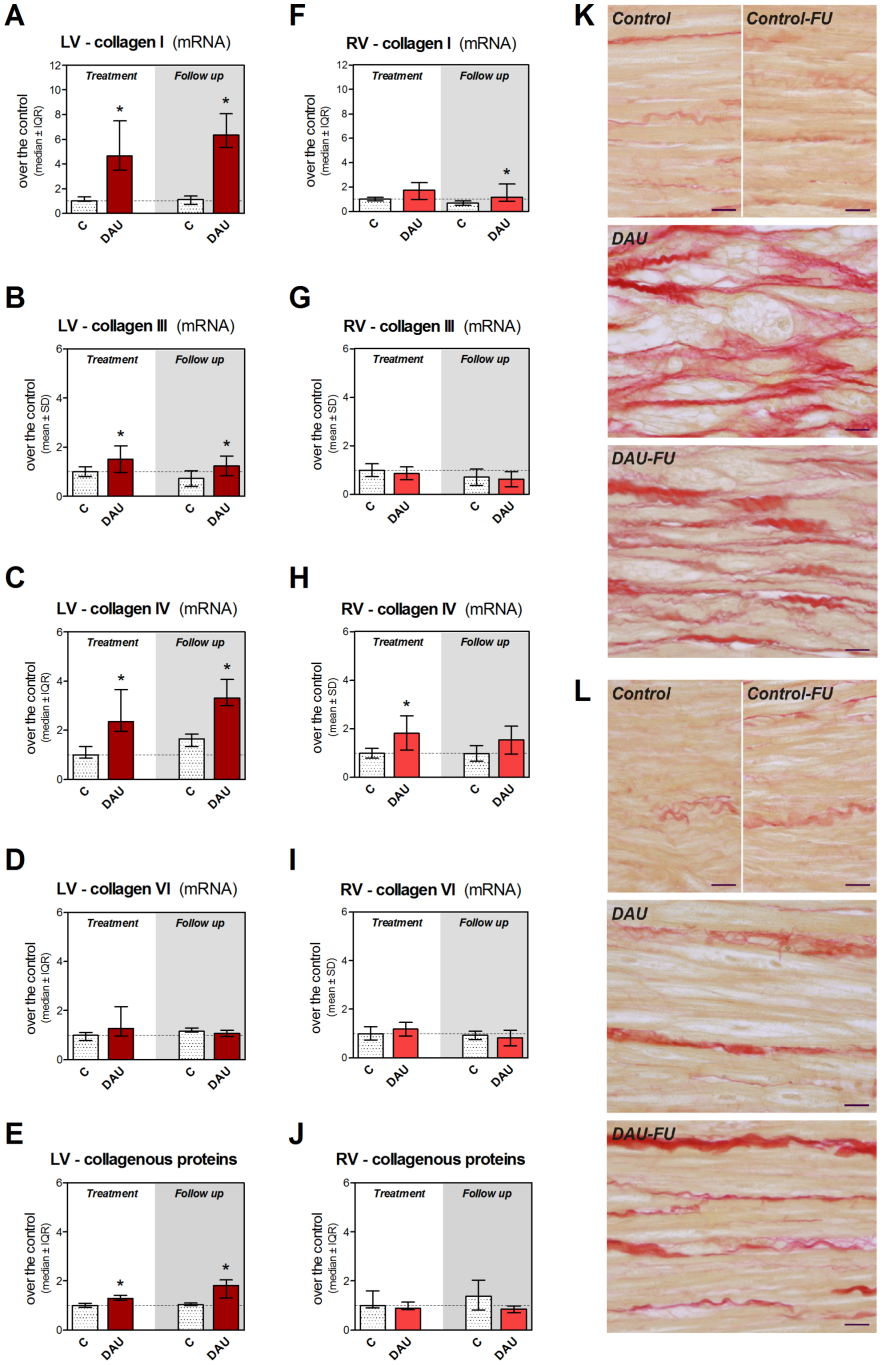
Extracellular matrix remodeling in chronic anthracycline cardiotoxicity and post-treatment follow up. Gene expression of collagen I (**A**, **F**), collagen III (**B**, **G**), collagen IV (**C**, **H**) and collagen VI (**D**, **I**) in the left and right ventricular myocardium, respectively. Biochemical analysis of collagenous proteins estimated by hydroxyproline determination in the left (**E**) and right (**J**) ventricular myocardium. Statistical significances (One Way ANOVA/One Way ANOVA on Ranks according to data character, *P*<0.05) within each study period (*). Detection of collagen fibers (particularly of collagen type I) using Picrosirius red staining in the left (**K**) and right (**L**) ventricular myocardium. Scale bar - 20 µm. C - control group, DAU - daunorubicin group, FU - follow up.

Further analyses showed profound DAU-induced changes of the extracellular matrix in the LV, while little to no change was found in the RV. This particularly concerned collagen I and IV (Fig. 11A and Fig. 11C) with 3- to 6-fold increased induction due to the treatment in both studied intervals, while collagen III showed only moderate increase in the LV myocardium (Fig. 11B) and no change was observed in collagen VI (Fig. 11D). With exception in collagen IV (Fig. 11H), gene expression of collagen proteins was not significantly altered by the treatment in the RV (Fig. 11F, Fig. 11G and Fig. 11I). Biochemical analysis of collagenous proteins confirmed significant increase due to the treatment in the LV (Fig. 11E), while no significant change was found in the RV (Fig. 11J). Picrosirius red staining documented markedly increased fibrotic tissue within the LV myocardium (Fig. 11K), particularly at the foci affected by advanced degenerative changes and this appeared even more accented in the post-treatment period where the collagenous fibers formed the scars. In contrast, the changes in the RV were much less frequent and rather isolated (Fig. 11L). Gene expression of the transforming growth factor beta 1 (TGFβ1), a crucial signaling molecule regulating the collagen expression pathway, was also found significantly up-regulated due to the treatment in the LV (Fig. S4a), while no significant change was found in the RV (Fig. S4b).

## Discussion

In this study, we induced the early-onset form of chronic ANT cardiotoxicity with congestive heart failure and dilated cardiomyopathy. We found virtually the same histopathological hallmarks as reported previously in animal and human hearts exposed to significant cumulative doses of ANT [5], [9]. In agreement with the previous reports [4], [7], [27], the degenerative changes were located mainly in the LV myocardium and interventricular septum, while the RV myocardium was markedly less affected. Besides characteristic LV dilation, some ANT-treated animals showed also dilation of the RV, but this was presumably a secondary event developing only after severe left-sided heart failure with marked lung congestion. The latter conditions suggested importance of pressure-overload for the development of distinct degenerative changes in the RV. Nevertheless, the overall histopathological picture in both ventricles remained markedly different even in animals suffering from end-stage cardiotoxicity.

Previous reports suggested that ANT-induced disarray and loss of myofibrils could be associated with the impaired expression of cardiac myofibrillar proteins [13], [14]. However, quantitative information about the expression of essential sarcomeric proteins in chronic ANT cardiotoxicity was missing. In this study, we have clearly documented perturbations in the expression of several key sarcomeric proteins. We report a severe and persisting down-regulation of cMLC1 expression due to the treatment which may explain decreased cMLC1 protein abundance determined previously in our 2D-DIGE study [28]. Since cMLC1 is known to be cleaved by caspase-3 [29], which we have previously found induced in our model [22], a role of enzymatic degradation also cannot be excluded. Cardiac MLC1 is an essential MLC isoform important for the stability of the myosin heads, appropriate cross-bridge cycle kinetics and force production [30], thus its persistent down-regulation in the LV may indeed be involved in the serious morphological and functional changes observed in our study. In contrast to *in vitro* experiments discussed above [14], we have not found significant change in MLC2v mRNA after ANT treatment, but we have determined a slight decrease in MLC2v protein abundance which suggests involvement of post-transcriptional regulation. Interestingly, the changes in gene expression and protein abundance of MLC isoforms were much less pronounced in the RV which corresponds with weaker toxic burden observed herein. We also show significant and persistent down-regulation of gene expression of both MHC isoforms in the LV after chronic ANT treatment. Previously, Bouček *et al.* [13] reported only non-significant alterations of βMHC mRNA levels in the LV after two doses of doxorubicin (1 mg/kg) in rabbits, but this treatment obviously could not induce significant cardiotoxicity. A significant drop of MHC proteins observed in the post-treatment period only indicates the rather weak and delayed effect of the transcriptional changes. De Beer *et al.* [31] reported a shift in abundance of MHC isoforms after doxorubicin treatment (2 mg/kg/week for 4 weeks) in rats, but we did not see such trend in our mRNA data. This discrepancy could be caused by potentially more complex relationship between the mRNA and protein levels of the isoforms. However, different severities of cardiotoxicity or interspecies differences may be also involved (*e.g.,* rats show inverse ratio of MHC isoforms as compared to both rabbits and humans [32]).

In contrast to thick filament proteins, gene expression of their thin filament counterparts (cardiac α-actin, troponin T and tropomyosin 1) was not altered in the LV myocardium and correspondingly, no change happened in cardiac α-actin at the protein level, despite evident morphological and functional changes being already in place. Some relative changes were seen in the post-treatment period, but even these were unlikely to explain the obvious difference in clinical phenotype. Indeed, we cannot exclude short-lasting impairment of expression of these genes after acute ANT exposure, but without significant persistence they can barely determine profound and irreversible loss of myofibrils in the LV. Our data are in agreement with previous study using the similar rabbit model (doxorubicin 2.5 mg/kg/week for 8 weeks), where no changes in cardiac α-actin expression were detected at mRNA level despite the development of cardiomyopathy [33]. It should be also noted that we have observed a certain tendency in control animals towards the increase in transcription of several sarcomeric and calcium handling proteins (particularly in the LV) in the post-treatment period. We assume that this change can be linked with the adaptation of the myocardium on some stress associated with regular experimental procedures continuing for an additional 10 weeks. However, this change was likely not distinct enough to be translated to the protein levels and change the phenotype. Interestingly, ANT-treated hearts did not share the same tendency which could implicate altered responses to this stress.

We have also demonstrated for the first time that titin expression is markedly and permanently down-regulated in the LV myocardium of ANT-treated rabbits, while this is not the case of the RV myocardium. Interestingly, the down-regulation of titin expression has been previously described in human dilated cardiomyopathy [34] which highlights the potential importance of this finding. ANT-induced alteration in titin was also found in the previous *in vitro* study [19], but these authors postulated calpain-mediated cleavage as the main mechanism instead. Although we have previously found increased calpain activity in the LV using the same model [28], a lack of raise of titin degradation product T2 argues against the dominant role of this hypothesis. Hence, the titin cleavage occurs only shortly after the ANT exposure or the change in titin abundance is transcriptionally regulated as suggested by our data. With respect to the central role of titin in sarcomere structure and function, these alterations may induce or significantly contribute to development of sarcomeric instability.

Transcriptional down-regulation of several key sarcomeric proteins (such as titin) in the LV may correspond with the recent *in vitro* work which links ANT-induced disarray/loss of myofibrils with down-regulation of transcriptional co−/factors GATA-4 and CARP [18]. Indeed, CARP can interact with titin to couple mechanical strain within the sarcomere to gene transcription of sarcomeric proteins [35] and it has been found rapidly down-regulated in isolated cardiomyocytes exposed to ANT [16], [18]. In contrast to these *in vitro* studies, we found distinct induction of CARP expression in the LV. This discrepancy might be determined by the chronic nature of our *in vivo* experiment and myocardial sampling one or more weeks after the treatment. Up-regulation can take place after drug elimination either as a compensatory reaction or due to the sarcomeric stress induced by the toxicity. Our data correspond with the previous *in vivo* study which also reported increased CARP expression after weekly doxorubicin (2 mg/kg) administration to piglets [36]. These authors also found a baseline asymmetry in CARP expression between both ventricles which may be of interest with respect to the propensity to development of cardiotoxicity. In addition, several *in vitro* studies have demonstrated that GATA-4 is down-regulated in cardiomyocytes after ANT exposure [15], [17], [18], [37], [38]. However, our study is first to show that this also happens in the LV of animals suffering from chronic ANT cardiotoxicity. It is also noteworthy that the RV is spared of this effect. Chen *et al.* [18] reported that in contrast to CARP overexpression, GATA-4 overexpression was able to rescue cardiomyocytes from ANT toxicity and myofibrillar disarray *in vitro*. This may correspond with the inability of CARP overexpression to overcome the development of cardiotoxicity in our study and it further highlights the potential mechanistic importance of persistent GATA-4 insufficiency.

Protein composition of the sarcomere and myofibrillar apparatus is subjected to dynamic physiological changes with the key role of the ubiquitin-proteasome system. In line with previous studies [20], [28], we have found significant induction of proteasome activity in the LV. In addition, we also show here that these changes are still in place in the post-treatment period and that only smaller ones occurred in the RV. Since there was marked difference in levels of polyubiquitinated proteins between LV and RV, it can be assumed that ubiquitin-proteasome system is unable to cope with the removal of damaged protein in the LV cardiomyocytes, while it may be relatively well functioning in the RV. Indeed, impaired ability of LV cardiomyocytes to remove damaged or misfolded proteins may further aggravate ANT-induced damage. Moreover, cardiac safety of combination of ANTs with proteasome inhibitors such as bortezomib (*e.g.,* in multiple myeloma treatment [39]) may warrant attention. In addition, impaired stability of myofibrils or their altered interaction with other organelles may trigger compensatory changes and further remodeling of the affected cardiomyocytes. We have confirmed marked up-regulation of desmin expression in the LV myocardium identified for the first time in our previous 2D-DIGE study [28]. We have clarified here that these alterations remain very marked even in the post-treatment period and that the RV is less affected. We assume primarily an adaptive role of desmin in this setting aiming to enhance structural stability of the damaged myofibrils, but further studies employing genetic manipulations should support this assumption.

Besides pathological remodeling of cardiomyocytes, ANT cardiotoxicity induced marked changes in non-myocyte cells and extracellular matrix. We have confirmed our previous 2D-DIGE finding of increased vimentin levels in the LV myocardium and present data suggest that this is likely a result of persisting up-regulation of gene transcription mainly in activated fibroblasts. Again smaller changes were recognized in the RV which was likely associated with less significant morphological damage. Using two independent histological techniques, we have described replacement fibrosis in the LV within the foci of cardiomyocytes affected by toxic damage and its further maturation in the post-treatment period, while this was not typically seen in the RV myocardium. Investigation of molecular events contributing to this process revealed increased expression of fibrillar collagens (mainly collagen I) presumably by activated fibroblasts. Reticular collagen IV, which is also expressed by cardiomyocytes [40], [41], was also markedly induced and thus we cannot exclude that even surviving cardiomyocytes could participate in efforts to strengthen the basal lamina. Unlike in our previous 2D-DIGE study [28], we have not found a change in collagen VI due to ANT treatment which could be potentially explained either by more complex relationship of mRNA and corresponding protein levels or by technical limitations of the previous proteomic approach. TGFβ in the LV myocardium may explain signaling leading to activation of fibroblasts and collagen I and III expression [42], [43] and a lack of TGFβ induction in the RV may be the reason for absence of the latter changes therein. However, our data contradict findings from a transcriptional analysis of chronic ANT cardiotoxicity in rats (doxorubicin 3 mg/kg/week for 12 weeks) which has surprisingly indicated down-regulation of gene expression of two collagen types (including important *COL3A1*) and intermediate filament vimentin in the LV [44]. Nevertheless, our data are in concert with overall morphological picture found in the LV.

The left-right asymmetry in molecular and morphological findings was likely translated to the change of cardiac function. This assumption was supported by a different expression of ANP which has been described as a myocardial marker of heart failure in rabbits [45]. The LV systolic dysfunction was severe and irreversible and it can be linked with loss and disarray of myofibrils, but the perturbation in expression of RYR2 might be also implicated. ANT-treatment also significantly disturbed LV relaxation which may be associated with impaired diastolic calcium handling particularly due to the lower availability of SERCA2a. The latter finding is in line with outcomes of a previous study [33]. Altered viscoelastic properties of the myocardium due to the increased collagen deposition could also be taken into account. It is noteworthy that the above described molecular events were weaker or almost absent in the RV myocardium. Furthermore, this particular data could explain ANT-induced calcium overload which we have documented in the LV in our previous study [46] and there might be also a link to degenerative changes.

The reason for the asymmetry in the response of the myocardium to ANT cardiotoxicity is unknown, but numerous differences might be implicated. The LV myocardium faces much higher mechanical workload which determines marked differences in myocardial morphology, mechanics, calcium handling, oxygen supply-demand profiles and oxidative phosphorylation as compared to the RV myocardium [47], [48]. However, we can only speculate whether the higher workload and related stress is crucial for triggering or clinical manifestation of ANT-induced molecular changes and thus it deserves further study. Interestingly, embryological studies have demonstrated that LV and RV cardiomyocytes originate from different heart fields each containing unique populations of cardiac progenitor cells and we cannot exclude that this may co-determine their different response to ANT cardiotoxicity [49], [50].

Indeed, interspecies differences may complicate translation of the experimental findings from animal models to human medicine, but employment of rabbits may offer several advantages as compared to commonly used rodents. Rabbit hearts are known to show considerably higher similarity to human hearts, particularly with respect to composition of sarcomere or function of calcium handling proteins [51]. While qualitatively similar findings might be also expectable in female rabbits, the present study used males only and thus, it remains to be determined whether females would show different propensity to development of these alterations. We have also described features associated with early-onset form of chronic ANT cardiotoxicity, but their translatability to late-onset form of the toxicity may not be straightforward and it should be tested on appropriate long-term animal models.

In conclusion, we have confirmed that the myocardium of both ventricles shows strikingly different propensity to the cardiotoxicity development. Furthermore, it is shown that the profound molecular remodeling concerns cardiomyocytes, other myocardial cells as well as extracellular matrix of the LV, while the majority of these changes are either distinctly weaker or completely absent in the RV. Marked down-regulation of expression of several sarcomeric proteins (mainly titin and thick filament components) may be connected with ANT-induced perturbation in stability and function of sarcomere in the LV. These changes may correspond with ANT-induced persistent down-regulation of expression of GATA-4 which was not found in the RV. These findings may be aggravated by insufficiency of ubiquitin-proteasome system in the LV which is not the case of the RV. The up-regulation of intermediate filaments is also more distinct in the LV and this likely reflects an effort to enhance stability of myofibrils (desmin) and activation of fibroblasts (vimentin). The latter finding then corresponds with the profound remodeling of the extracellular matrix which happened almost exclusively in the LV. Hence, the present study sheds new light on the molecular aspects of remodeling of the LV and RV in chronic ANT cardiotoxicity and together with further investigations it may provide important insights into the molecular basis of these serious complications in the cancer chemotherapy.

## Supporting Information

Figure S1 **Heart to body weight ratio in chronic anthracycline cardiotoxicity and post-treatment follow up.** Statistical significances (One Way ANOVA, *P*<0.05) within each study period (*). C - control group, DAU - daunorubicin group.
(PDF)

Figure S2 **Expression of tropomyosin 1 in chronic anthracycline cardiotoxicity and post-treatment follow up.** Gene expression in the left (**a**) and right (**b**) ventricular myocardium, respectively. Statistical significances (One Way ANOVA/ANOVA on Ranks according to data character, *P*<0.05) within each study period (*). C - control group, DAU - daunorubicin group.
(PDF)

Figure S3 **Activity of proteasome system in chronic anthracycline cardiotoxicity and post-treatment follow up.** Changes in activity of proteasome chymotrypsin-like (**a**, **d**), trypsin-like (**b**, **e**) and PGP-like activities (**c**, **f**) in the left and right ventricular myocardium, respectively. Statistical significances (One Way ANOVA/ANOVA on Ranks according to data character, *P*<0.05) within each study period (*). C - control group, DAU - daunorubicin group.
(PDF)

Figure S4 **Expression of transforming growth factor beta 1 in chronic anthracycline cardiotoxicity and post-treatment follow up.** Gene expression in the left (**a**) and right (**b**) ventricular myocardium, respectively. Statistical significances (One Way ANOVA/ANOVA on Ranks according to data character, *P*<0.05) within each study period (*). TGFβ1 - transforming growth factor beta 1, C - control group, DAU - daunorubicin group.
(PDF)

Table S1 **Summary of gene expression assays used for quantitative real-time PCR.**
(DOCX)

Table S2 **Protein identification by mass spectrometry.** The protein bands were cut from Coomassie blue-stained gels and analyzed by nanoHPLC-Q-TOF as specified in Method S1. Obtained spectra were searched against the rabbit protein database downloaded from NCBI using MASCOT search engine. Unambiguously identified proteins are listed below along with the accession number, protein score, number of peptide matches, molecular weight and short description.
(DOCX)

Method S1 (DOCX)

Method S2 (DOCX)
