# Supplementary material for: Molecular Remodeling of Left and Right Ventricular Myocardium in Chronic Anthracycline Cardiotoxicity and Post-Treatment Follow Up
Source: PLoS One. 2014 May 7;9(5):e96055. doi: 10.1371/journal.pone.0096055 (PMC4013127; doi:10.1371/journal.pone.0096055)
Supplement: Figure S1 — Heart to body weight ratio in chronic anthracycline cardiotoxicity and post-treatment follow up. Statistical significances (One Way ANOVA, P<0.05) within each study period (*). C - control group, DAU - daunorubicin group. (PDF) [file pone.0096055.s001.pdf]

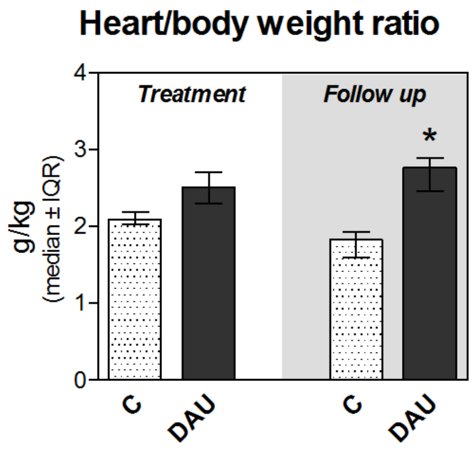


**Figure S1. Heart to body weight ratio in chronic anthracycline cardiotoxicity and post-treatment follow up.** Statistical significances (One Way ANOVA, $P<0.05$) within each study period (*). C - control group, DAU - daunorubicin group.
