# Supplementary material for: Molecular Remodeling of Left and Right Ventricular Myocardium in Chronic Anthracycline Cardiotoxicity and Post-Treatment Follow Up
Source: PLoS One. 2014 May 7;9(5):e96055. doi: 10.1371/journal.pone.0096055 (PMC4013127; doi:10.1371/journal.pone.0096055)
Supplement: Figure S4 — Expression of transforming growth factor beta 1 in chronic anthracycline cardiotoxicity and post-treatment follow up. Gene expression in the left (a) and right (b) ventricular myocardium, respectively. Statistical significances (One Way ANOVA/ANOVA on Ranks according to data character, P<0.05) within each study period (*). TGFβ1 - transforming growth factor beta 1, C - control group, DAU - daunorubicin group. (PDF) [file pone.0096055.s004.pdf]

a

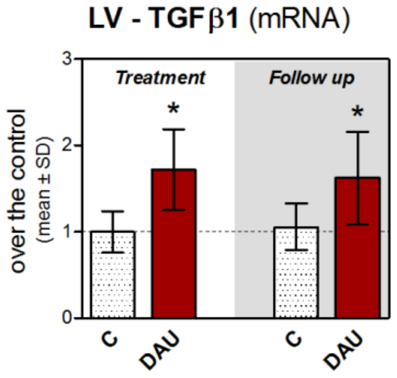

b

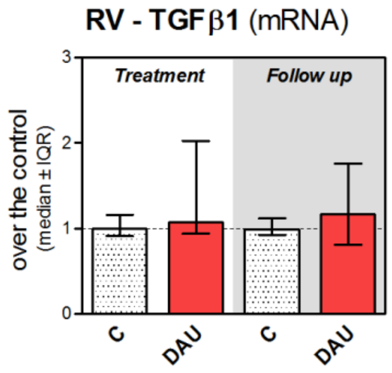

Figure S4. Expression of transforming growth factor beta 1 in chronic anthracycline cardiotoxicity and post-treatment follow up. Gene expression in the left (**a**) and right (**b**) ventricular myocardium, respectively. Statistical significances (One Way ANOVA/One Way ANOVA on Ranks according to data character, $P<0.05$) within each study period (*). TGFβ1 - transforming growth factor beta 1, C - control group, DAU - daunorubicin group.
