# Supplementary material for: Molecular Remodeling of Left and Right Ventricular Myocardium in Chronic Anthracycline Cardiotoxicity and Post-Treatment Follow Up
Source: PLoS One. 2014 May 7;9(5):e96055. doi: 10.1371/journal.pone.0096055 (PMC4013127; doi:10.1371/journal.pone.0096055)
Supplement: Table S1 — Summary of gene expression assays used for quantitative real-time PCR. (DOCX) [file pone.0096055.s005.docx]

**Table S1. Summary of gene expression assays used for quantitative real-time PCR.**

| Gene name | Gene symbol | # | qPCR assay | Sequence for design | Localization |
| --- | --- | --- | --- | --- | --- |
| *Cardiac α- actin* | *ACTC* | GB | ocACTC_Q1 | XM_002717998.1 | exon1 / exon2 |
| *Ankyrin repeat domain 1, cardiac* | *ANKRD1* | AB | oc03396975_m1 | NM_001082054.1 | exon5 / exon6 |
| *Natriuretic peptides A* | *NPPA* | AB | Oc03397714_g1 | NP_001075731.1 | exon1 / exon2 |
| *Collagen type I α 1* | *COL1A1* | GB | ocCOL1A1_Q1 | AY633663 | exon48 / exon49 |
| *Collagen type III α 1* | *COL3A1* | GB | ocCOL3A1_Q2 | XM_002712333.1 | exon47 / exon48 |
| *Collagen type IV α 2* | *COL4A2* | GB | ocCOL4A2_Q2 | XM_002712951.1 | exon40 / exon41 |
| *Collagen type VI α 1* | *COL6A1* | GB | ocCOL6A1_Q2 | XM_002723433.2 | exon13 / exon15 |
| *Desmin* | *DES* | GB | ocDES_Q1 | DP001041 | exon6 / exon9 |
| *GATA-4 transcription factor* | *GATA4* | AB | oc04096744_mH | AJ291310.1 | exon1 / exon2 |
| *Hypoxanthine guanine phosphoribosyl transferase 1* | *HPRT1* | GB | ocHPRT1_Q3 | NM_001105671 | exon6 / exon7 |
| *Myosin heavy chain isoform α, cardiac* | *MYH6* | GB | ocMYH6_Q1 | XM_002717908.1 | exon14 / exon16 |
| *Myosin heavy chain isoform β, cardiac* | *MYH7* | GB | ocMYH7_Q1 | XM_002718084.1 | exon14 / exon16 |
| *Myosin light chain isoform 2* | *MYL2* | GB | ocMYL2_Q1 | XM_002719750.1 | exon2 / exon3 |
| *Myosin light chain isoform 1* | *MYL3* | GB | ocMYL3_Q1 | XM_002713321.1 | exon4 / exon5 |
| *Sodium/calcium exchanger* | *SLC8A1* | AB | Oc04250277_m1 | NM_001170958.1 | exon6 / exon7 |
| *Ryanodine receptor 2* | *RYR2* | AB | Oc03398565_m1 | NM_001082757.1 | exon55 / exon56 |
| *Sarcoplasmic/endoplasmic reticulum calcium ATPase 2* | *ATP2A2* | AB | Oc04096460_m1 | NM_001089321.1 | exon7 / exon8 |
| *Transforming growth factor β1* | *TGFB1* | AB | oc04176122_u1 | AB020217.1 | exon3 |
| *Tropomyosin 1* | *TMP1* | AB | oc03399484_m1 | NM_001105688.1 | exon2 / exon3 |
| *Troponin T, cardiac* | *TNNT2* | AB | oc03396241_m1 | L40178.1 | NA |
| *Titin* | *TTN* | AB | oc03396431_m1 | U28657.1 | exon2 / exon3 |
| *Vimentin* | *VIM* | GB | ocVIM_Q1 | XM_002717420.1 | exon1 / exon3 |

^#^Individual gene expression assays were obtained from Generi Biotech (GB, Hradec Králové, Czech Republic) or Applied Biosystems (AB, Foster City, CA).
