# Supplementary material for: Molecular Remodeling of Left and Right Ventricular Myocardium in Chronic Anthracycline Cardiotoxicity and Post-Treatment Follow Up
Source: PLoS One. 2014 May 7;9(5):e96055. doi: 10.1371/journal.pone.0096055 (PMC4013127; doi:10.1371/journal.pone.0096055)
Supplement: Table S2 — Protein identification by mass spectrometry. The protein bands were cut from Coomassie blue-stained gels and analyzed by nanoHPLC-Q-TOF as specified in Method S1. Obtained spectra were searched against the rabbit protein database downloaded from NCBI using MASCOT search engine. Unambiguously identified proteins are listed below along with the accession number, protein score, number of peptide matches, molecular weight and short description. (DOCX) [file pone.0096055.s006.docx]

**Table S2. Protein identification by mass spectrometry**.

The protein bands were cut from Coomassie blue-stained gels and analyzed by nanoHPLC-Q-TOF as specified in Method S1. Obtained spectra were searched against the rabbit protein database downloaded from NCBI using MASCOT search engine. Unambiguously identified proteins are listed below along with the accession number, protein score, number of peptide matches, molecular weight and short description.

| Band | Accession | MASCOT score | Matches | Mass | Description |
| --- | --- | --- | --- | --- | --- |
| 01 | gi\|291403583 | 1169 | 34 | 222 949 | myosin, heavy chain 7, cardiac muscle, beta [Oryctolagus cuniculus] |
| 02 | gi\|291391832 | 115 | 3 | 3 708 013 | titin [Oryctolagus cuniculus] |
