# Supplementary material for: Molecular Remodeling of Left and Right Ventricular Myocardium in Chronic Anthracycline Cardiotoxicity and Post-Treatment Follow Up
Source: PLoS One. 2014 May 7;9(5):e96055. doi: 10.1371/journal.pone.0096055 (PMC4013127; doi:10.1371/journal.pone.0096055)
Supplement: Method S1 — (DOCX) [file pone.0096055.s007.docx]

**Method S1**

**In-gel digestion of proteins**

The bands of interest from acrylamide gel strengthened with agarose gel were excised, shortly rinsed in water at room temperature, crushed into pieces and destained in 50% acetonitrile at 30 °C for 30 min, followed by a short rinsing in acetonitrile. The disulfide bonds were reduced by adding 50 µL of 5 mM tris(2-carboxyethyl)phosphine (Pierce, Rockford, IL) and incubating for 60 min at 60 °C. Reduced cysteine residues were thiomethylated in 50 µL of 10 mM methyl methanethiosulfonate (Pierce, Rockford, IL) for 10 min at room temperature. Gel pieces were then equilibrated in 25 mM NH_4_HCO_3_, rinsed in 25 mM NH_4_HCO_3_ in 50% acetonitrile and vacuum-dried. Dried gel pieces were rehydrated in 10 µL of 10 mM acetic acid containing 400 ng of sequencing grade trypsin (Promega, Madison, WI) for 10 min and then covered by additional 30 µL of 25 mM NH_4_HCO_3_. Digestion was performed overnight at 37 °C. Generated peptides were extracted by adding of 30 µL of 80% acetonitrile with 0.1% TFA three times. Extracts were vacuum-dried, dissolved in 5% acetonitrile with 0.1% TFA and desalted on Empore C18-SD SPE cartridges (Sigma, St. Louis, MO). Desalted peptides were vacuum-dried and dissolved in 25 µL of 5% acetonitrile with 0.1% TFA.

**LC-MS analysis and protein identification**

For each sample, 4 µL of dissolved peptides was injected into the CapLC system (Waters, Manchester, UK) and trapped on NanoEase Trap Column 180 µm x 23 mm filled with Atlantis dC18, 5 µm (Waters, Manchester, UK). The peptides were separated on the analytical column NanoEase 75 µm x 150 mm Atlantis dC18, 3 µm (Waters, Manchester, UK) by a gradient formed by mobile phase A (2% acetonitrile with 0.1% formic acid) and mobile phase B (98% acetonitrile with 0.1% formic acid) running from 5% to 50% of mobile phase B in 20 min at a flow rate of 0.3 µL/min at 40 °C. The LC system was connected to a Q-TOF Ultima API (Waters, Manchester, UK). Data-directed acquisition was recorded for each injection. Obtained spectra were searched against the rabbit protein database downloaded from NCBI using MASCOT search engine.
