# Supplementary material for: Molecular Remodeling of Left and Right Ventricular Myocardium in Chronic Anthracycline Cardiotoxicity and Post-Treatment Follow Up
Source: PLoS One. 2014 May 7;9(5):e96055. doi: 10.1371/journal.pone.0096055 (PMC4013127; doi:10.1371/journal.pone.0096055)
Supplement: Method S2 — (DOCX) [file pone.0096055.s008.docx]

**Method S2**

**Measurement of proteasome activity**

Proteasome chymotrypsin, trypsin and peptidyl-glutamyl peptidase (PGP)-like activities were determined in the LV and RV using commercial kit (Proteasome-Glo 3-Substrate System, Promega, Madison, WI) according to the manufacture’s protocol. Frozen heart tissue (approx. 50 mg) was homogenized in 10 mM HEPES buffer (pH 7.6) for 20 min and then centrifuged at 16 000 x g for 10 min, all at 4 °C. After centrifugation, the supernatants were collected for further analysis of proteasome activities. Luciferin Detection Reagent was suspended with Proteasome-Glo Buffer and supplemented with following to prepare reagents for measurement of chymotrypsin, trypsin and PGP-like activities, respectively: 40 μM Suc-LLVY-aminoluciferin, 30 μM Z-LRR-aminoluciferin and 40 μM Z-nLPnLD aminoluciferin. Each reagent was incubated 60 min at the room temperature to remove residual free aminoluciferin prior to use. After protein-concentration determination, each sample was diluted with 10 mM HEPES buffer enriched with 2 mM ATP and 5 mM Mg^2+^ to the final protein concentration 0.1 mg/mL. An equal volume of luminescent proteasome reagent containing the appropriate aminoluciferin substrate was added to the diluted sample mixture and incubated at room temperature for 30 min. The luminescence was measured with Tecan Infinite 200 (Tecan Group, Männedorf, Switzerland). The luminescence units were normalized on protein content in each sample. The fold-increase in proteasome activities was determined by comparing these results in the control group. All assays were performed in triplicate.
